# Dimensions of women’s empowerment on access to skilled delivery services in Nepal

**DOI:** 10.1186/s12884-020-03309-9

**Published:** 2020-10-15

**Authors:** Januka Khatiwada, Basilua Andre Muzembo, Koji Wada, Shunya Ikeda

**Affiliations:** 1 Department of Public Health, School of Medicine, International University of Health and Welfare, 4-3 Kozunomori, Narita City, Chiba 286-8686 Japan; 2 Graduate School of Medicine, Dentistry and Pharmaceutical Sciences, Okayama University, Okayama, Japan

**Keywords:** Skilled delivery service, women’s empowerment, Demographic and health survey, Nepal

## Abstract

**Background:**

Each day, approximately 810 women die during pregnancy and childbirth and 94% of the deaths take place in low and middle income countries. Only 45% of the births in South Asia are attended by skilled professionals, which is lower than that in other Asian regions. Antenatal and postnatal care received from skilled providers can help prevent maternal and neonatal mortality by identifying pregnancy-related complications. Women’s empowerment is considered to be a significant determinant of maternal health care outcomes; however, studies on the contextual influences of different dimensions of empowerment in Nepal are relatively limited. Therefore, this study analyzed nationwide survey data to examine the influence of women’s economic empowerment, sociocultural empowerment, familial/interpersonal empowerment and media and information technology empowerment on accessing skilled delivery services among the married women in Nepal.

**Methods:**

This study examined the influence of women’s empowerment on skilled delivery services among married women (*n* = 4400) aged 15–49 years using data from the 2016 Nepal Demographic and Health Survey. Descriptive analysis and binary logistic regression analysis were employed to analyze the data.

**Results:**

Significant associations were found between women’s media and information technology empowerment, economic empowerment and sociocultural empowerment and access to skilled birth attendants. Specifically, the education of women, their occupation, owning a bank account, media exposure, and internet use were significantly associated with the use of skilled birth attendants.

**Conclusion:**

Focusing on women’s access to media and information technology, economic enhancement and education may increase the use of skilled birth attendants in Nepal.

## Background

Each day, approximately 810 women die during pregnancy and childbirth and 94% of these deaths take place in low and middle income countries [1]. Only 45% of the births in South Asia are attended by skilled birth attendants (SBAs), which is lower than that in other Asian regions [2]. Antenatal care (ANC) and postnatal care received from skilled providers can help prevent maternal and neonatal mortality by identifying pregnancy-related complications [3]. Despite the formulation and implementation of programs related to birth preparedness, complication readiness and national safe motherhood to increase the share of deliveries assisted by skilled professionals by the government of Nepal [4], only 57.4% of the births in the country occur in the presence of skilled health care providers [5]. Due to low rate of women’s literacy (58%) [6], the lack of sufficient health facilities and access difficulties, the share of deliveries attended by skilled professionals is still minimal. The factors associated with a lower rate of skilled deliveries reported by other studies are the following: lack of education, poverty, inadequate antenatal care visits, pregnancy complications and a lack of women’s empowerment [7–9].

Women’s empowerment should be viewed as one of the dimensions of maternal health since it plays significant role in the health and well-being of women, children and the family. It has also been one of the major UN Sustainable Development Goals (SDGs) [10]. The influence of women’s empowerment on access to skilled delivery service has already been associated with maternal health services [11–15]. Existing studies have documented the significant association between women’s empowerment and maternal health service utilization [11, 16–21].

However, what explicitly measures women’s empowerment in different contexts and cultural backgrounds is still controversial. Some researchers have employed education, economic status, household decision making and some other variables such as attitude towards gender-based violence and support from family and kin to measure the influence of women’s empowerment on access to maternal health care services [13, 18, 20–22]. Different dimensions of women’s empowerment might be independently effective in different contexts and cultural backgrounds such as in Africa, Asia and other countries [14, 15, 23]. There are many studies that have analyzed the influence of women’s empowerment on access to skilled delivery services using the data from the Demographic and Health Survey (DHS) collected for different countries [15, 18, 20, 23]. These studies have focused on the individual, family and community level effects on maternal health care services [23, 24], whereas some have categorized women’s empowerment using labor force participation, household decision making, family planning and education [13, 17]. However, relatively less attention has been given to the different dimensions of empowerment that may have different levels of effectiveness in different contexts. For instance, the economic prosperity of women in lower and high income countries might play different roles in pushing women to obtain skilled maternal health services. Despite having a good economic status, the existence of stereotypical beliefs and norms underpinned as shame, stigmatization, and privacy matters may hinder women in obtaining SBAs in poor countries such as Nepal where various kinds of traditional norms and beliefs are highly dominant. Therefore, this study analyzed the nationwide survey data to examine the influence of women’s economic empowerment, sociocultural empowerment, familial/interpersonal empowerment and media and information technology empowerment on accessing skilled delivery services among the married women aged 15–49 years in Nepal.

## Conceptual framework

There are some indicators that have been used to measure county-level empowerment and gender equality status. The indicators include the Gender Development Index (GDI), which measures the status of men and women based on life expectancy; educational status; per capita income; and the Gender Inequality Index (GII), which is used to measure reproductive health, educational status, political participation in parliament and labor force participation [25]. These indicators superficially measure the national level dimensions and not the individual level dimensions, which mean that they lack the cultural aspects of empowerment.

Theoretically, women’s empowerment is defined as a process [26–28] and as a goal [29] . Kabeer has divided women’s empowerment in three broad categories, namely, 1. Agency, which defines the decision making power regardless of the power relation; 2. Resources, which includes the health education and physical assets that lead agency to be executed; and 3. Achievements, which are the economic opportunities and improved sociopolitical status considered as an outcome of agency [26]. Most of the authors have used Kabeer’s concept of agency and resources to analyze the data pertaining to women’s empowerment [14, 17–19, 21]. Malhotra summarized the existing literatures categorizing women’s empowerment into Economic, Sociocultural, Familial/Interpersonal, Legal, Political and Psychological dimensions [30].

**Fig. 1:**
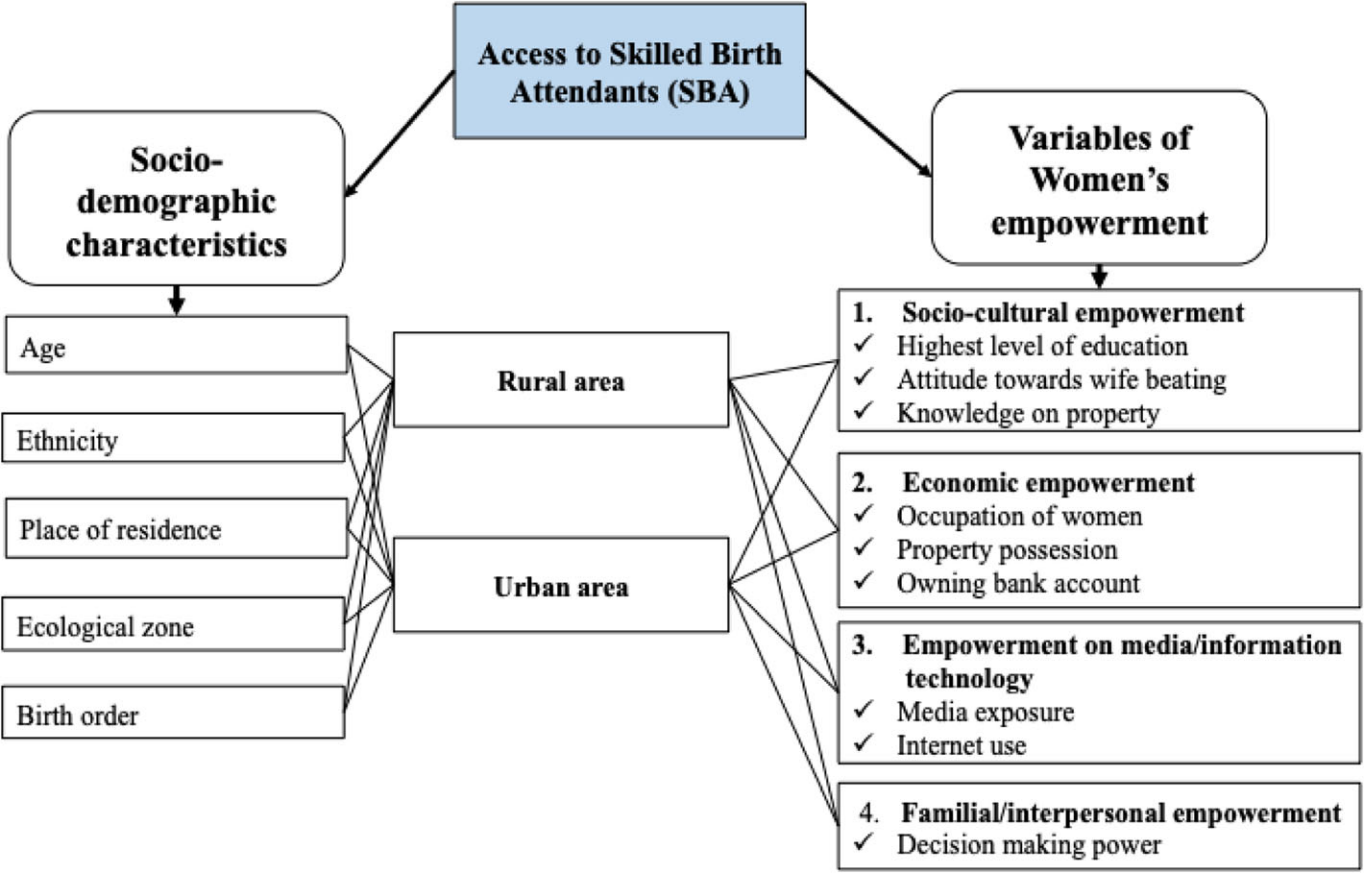
Conceptual framework

By reviewing the abovementioned literature on women’s empowerment, we generated a conceptual framework including the sociodemographic background and dimensions of women’s empowerment. Women’s empowerment has been divided into different dimensions such as sociocultural, economic and familial/interpersonal empowerment with reference to Malhotra [30] and Kabeer [26]. We also categorized media and information technology empowerment because the DHS used media exposure and ownership of a bank account as dimension of empowerment in Nepal [4, 31] (Fig. 1).

## Methods

This is a cross-sectional study of a national representative sample collected by the Nepal Demographic and Health Survey (NDHS), 2016. We conducted secondary data analysis by limiting the women of reproductive age of 15–49 as having given birth in the 5 preceding years. The reproductive age was selected following the WHO’s guidelines [32]. We also precisely selected the variables related to empowerment including only the individual information of women.

### Sampling technique

The NDHS applied an updated version of the sampling technique of the National Population and Housing Survey (NPHS) conducted by the Central Bureau of Statistics (CBS), 2011. The survey used two-stage stratified cluster sampling in rural areas and three-stage cluster sampling in urban areas. In rural areas, wards were selected as the Primary Sampling Units (PSUs), and households were selected from the sample PSUs. In urban areas, a three-stage selection sampling design was used, where the wards were selected as the Primary Sampling Units (PSUs) and an EA was selected from each PSU. Then, households were selected from the sample EAs.

The provinces were stratified into urban and rural areas, yielding 14 sampling strata. Samples of wards were selected independently in each stratum in two stages. Implicit stratification and proportional allocation were achieved at each of the lower administrative levels by sorting the sampling frame within each sampling stratum before sample selection, according to the administrative units in different levels, and by using a probability proportional-to-size selection in the first stage of sampling (URL: https://www.dhsprogram.com/Publications).

### Study participants and sample size

All married women aged 15–49 years who were either permanent residents of the selected households or visitors who stayed in the households the night before the survey were eligible to be interviewed by the DHS. In the interviewed households, 13,089 women aged 15–49 years were identified for individual interviews; and interviews were completed with 12,862 women, yielding a response rate of 98% [4]. For this study, the final sample was 4400 women who were currently married and had live birth in the 5 years preceding the survey.

(URL:https://www.dhsprogram.com/data/dataset/Nepal_Standard-DHS_2016. cfm?)

### Data collection tools and techniques

The NDHS applied face-to-face interviews using a structured questionnaire with the eligible women to collect data. The questionnaire was developed and finalized in English following the standard MEASURE DHS program guidelines [33] and the questionnaires were translated into Nepali and other local languages. The questionnaire was pretested by trained experienced enumerators.

### Data analysis technique

We weighted the sample before performing the data analysis. The sample domain and cluster design were also prepared, creating complex sample analysis (CSA). The descriptive analysis includes frequencies and percentages along with the Pearson Chi square test to determine the difference in SBA utilization according to the background characteristics. The level of significance was *p* < 0.05. We used binary logistic regression analysis to measure the association between SBAs and other independent variables. We presented the results in odds ratios (ORs), 95% confidence intervals (CIs) and *p* value p < 0.05. We analyzed the data using IBM SPSS statistics ver. 25.

### Ethical considerations

The data were collected for Nepal Demographic and Health Survey 2016 following the standard MEASURE DHS guidelines. The survey protocol was approved by Nepal Health Research Council (NHRC) (reg. no. 329/2015) and the ICF Institutional Review Board Calverton, Maryland, USA (ICF project number 132989.0.000.NP.DHS.01). NDHS 2016 obtained written consent from the household head to carry out the interviews. The survey data can be obtained from the ICF upon their request.

### Definition of variables

#### Outcome variables

The outcome variable for this study is a binary response regarding whether the women delivered their child using skilled attendants. Birth assistance was recoded as a dichotomous variable with a value for 0 for skilled attendants (doctors, nurses and midwifes) and 1 for unskilled attendants (health assistance, MCH worker, village health workers, traditional birth attendant, female community health volunteer, relative/friends/neighbors, and others).

### Exposure variables

The exposure variables have been selected based on their theoretical and empirical significance and the objective of our analysis, as shown in the given Table 1.

**Table 1:** Categorization of exposure variables

Age	Assigned a value of 0 for below 25, 1 for 26 to 30 and 2 for above 31.
Ethnicity	Described in 4 categories as follows: a value of 3 for Brahmin/kshetri, which included hill Brahmin, hill Kshetri, and Terai Brahmin Kshetri; 2 for Janajati, including Newar, Hill Janajati, and Terai Janajati; 1 for Dalit, which included Hill Dalit and Terai Dalit; and 0 for Other castes, which included the Other Terai caste group, Muslim and Others.
Type of place of residence	Coded as 0 for urban and 1 for rural.
Ecological zone	Coded as 0 for mountain, 1 for hill and 2 for Terai (plain).
Birth order	Coded as 0 for birth order 1, 1 for birth orders 2–3, 2 for birth orders 4–5 and 3 for birth order 6 + .
Education	Assigned a value of 0 for no education, 1 for primary, 2 for secondary and 3 for higher education.
Attitude towards wife beating	Attitude towards wife beating was based on the responses to four DHS questions asking whether the husband was justified in wife beating if she goes out without telling him, neglects the children, argues with him, or burns food. We assigned a value of 0 for a negative attitude if a woman says no for all the questions and 1 for a positive attitude if a woman says yes for all the questions.
Knowledge of property	If a respondent answered no regarding knowing how much property they have and whose name it is registered in, it is assigned a value of 0; and it is assigned a value of 1 if the respondent answered yes to knowing how much property they have and who owns that property.
Occupation of women	Coded as 0 for not working, 1 for Agriculture self-employed and 2 for other occupations including Professional/technical/managerial, Clerical, Sales/services, skilled manual labor, and unskilled manual labor.
Property possession	A binary variable that is assigned a value of 0 if a respondent does not own any land or a house and 1 if the respondent owned 1 or 2 properties.
Owns a bank account	A binary response that was 0 for no and 1 for yes.
Media exposure	A composite variable combining whether a respondent reads the newspaper or a magazine, listens to the radio and watches TV with a value of 0 if a woman lacks access to all the three media; 1 for medium access if a woman has access to one of the three forms of media; and 2 for high access to media if a woman has access to more than one of the forms of media at least once a week.
Internet use	Coded as 0 for never and 1 for sometimes.
Decision making	Decision making autonomy was based on the question asked to women in the DHS regarding who makes decisions on the respondent’s health, larger household purchases and visits with family or relatives. A respondent who made more than one decision, either alone or jointly with her husband, was coded 1 as having high decision-making authority; and if respondent made none of the decisions either alone or jointly with her husband, it was coded as 0 as low decision making authority.

## Results

Table 2 shows the summary of the characteristics of the women who were assisted in their deliveries by skilled and unskilled birth attendants. The mean (SD) age of the women who were assisted in their deliveries by both skilled and unskilled attendants was 26.1 (0.12) years. There was a significant difference (*p* < 0.05) between those having skilled and unskilled attendants among all the variables studied, except for ecological zone (*p* = 0.131) and women’s knowledge of property (*p* = 0.689). The women belonged to Brahmin/Kshetri (28.7%), Janajati (32.8%), Dalit (12.8% and other castes (25.7%). The percentage of women living in urban areas was higher (53.8%) than that living in rural areas (44.2%). Most of the women were from Terai (53.6%) than Hills (39.3%) and Mountains (7.1%). There were more women that had a second or third birth order (43.6%) than those with a first order birth (42.1%). Fewer women had higher education (15.1%) and most of the women (71.5%) had negative attitudes towards wife beating. As many as 94.2% of women knew who owned the property and the majority (90.8%) did not own property in their name. A majority of women (63.7%) did not have a bank account. The media exposure among the women was high (15.3%), medium (39.9%) or low (44.7%).

**Table 2:** Baseline characteristics of the study population and univariable association of the exposure variables with the outcome variables

Variables	Weighted Frequency (%) (***N*** = 4400)	Delivery assisted by skilled Professionals (%) (***N*** = 2353)	Delivery assisted by unskilled Professionals (%) (***N*** = 2047)	***P*** value for X2 test
**Age**				
Below 25	2243 (51.0)	1276 (29.0)	967 (22.0)	0.006
26 to 30 years	1376 (31.3)	695 (15.8)	682 (15.5)	
31 and above	781 (17.8)	382 (8.7)	399 (9.1)	
**Ethnicity**				
Other Caste	1129 (25.7)	583 (13.3)	546 (12.4)	0.018
Dalit	565 (12.8)	261 (5.9)	304 (6.9)	
Janajati	1445 (32.8)	759 (17.2)	686 (15.6)	
Brhamin and Kshetri	1261 (28.7)	750 (17.0)	512 (11.6)	
**Type of place of residence**				
Urban	2456 (53.8)	1497 (34.0)	959 (21.8)	0.000
Rural	1944 (44.2)	856 (19.4)	1088 (24.7)	
**Ecological zone**				
Mountain	313 (7.1)	128 (2.9)	186 (4.2)	0.131
Hill	1728 (39.3)	931 (21.2)	979 (18.1)	
Terai	2359 (53.6)	1294 (29.4)	1065 (24.2)	
**Birth order**				
1	1865 (42.4)	1228 (27.9)	637 (14.5)	0.000
2–3	1919 (43.6)	939 (21.3)	980 (22.3)	
4–5	457 (10.4)	135 (3.1)	321 (7.3)	
6+	160 (3.6)	50 (1.1)	110 (2.5)	
**Women’s empowerment**				
**Socio-cultural Empowerment**				
**Highest educational level**				
No education	1384 (31.5)	508 (11.5)	876 (19.9)	0.000
Primary	856 (19.5)	404 (9.2)	452 (10.3)	
Secondary	1502 (34.1)	936 (21.3)	566 (12.9)	
Higher	658 (15.0)	505 (11.5)	153 (3.5)	
**Attitude towards wife beating**				
Negative attitude	3148 (71.5)	1740 (39.5)	1408 (32.0)	0.012
Positive attitude	1253 (28.5)	613 (13.9)	640 (14.5)	
**Knowledge on property**				
Unknown	253 (5.8)	139 (3.2)	114 (2.6)	
Known	4147 (94.2)	2214 (50.3)	1933 (43.9)	0.689
**Economic empowerment**				
**Occupation of Women**				
Not working	1811 (41.2)	1035 (23.5)	777 (17.7)	0.000
Agriculture	1955 (44.4)	889 (20.2)	1066 (24.2)	
Other occupations	634 (14.4)	429 (9.8)	205 (4.7)	
**Property possession**				
Not owned	3994 (90.8)	2082 (47.3)	1912 (43.5)	
Owned	406 (9.2)	270 (6.1)	136 (3.1)	0.000
**Owns bank account**				
No	2801 (63.7)	1347 (30.6)	1454 (33.0)	
Yes	1599 (36.3)	1005 (22.8)	594 (13.5)	0.000
**Empowerment on media/information technology**				
**Media exposure**				
No access	1967 (44.7)	814 (18.5)	1152 (26.2)	
Medium access	1757 (39.9)	1046 (23.8)	710 (16.1)	0.000
High access	677 (15.3)	492 (11.2)	185 (4.2)	
**Internet use**				
Never	3516 (79.9)	1669 (37.9)	1847 (42.0)	
Sometimes	884 (20.1)	684 (15.5)	200 (4.6)	0.000
**Familial/Interpersonal empowerment**				
**Decision making**				
Low	2438 (55.4)	1252 (28.5)	1186 (26.9)	0.016
High	1962 (44.6)	1100 (25.0)	862 (19.6)	

Table 3 presents the results of the logistic regression analysis. The confounding factors used for the analysis were age, ethnicity, type of residence, ecological zone, and birth order.

**Table 3:** Crude and adjusted Odds Ratios of skilled birth attendance using a logistic regression with complex sampling analysis (*n* = 4400)

Variables	Crude OR (95%CI)	Adjusted OR (95%CI)	***P*** value
**Confounding factors**			
**Age**			0.024
Below 25	1	1	
26 to 30 years	0.77 (0.63 to 0.94)	0.96 (0.73 to 1.26)	
31 and above	0.73 (0.59 to 0.90)	1.49 (1.09 to 2.03)	
**Ethnicity**			0.087
Other Caste	1	1	
Dalit	0.81 (0.57 to 1.13)	0.87 (0.59 to 1.28)	
Janajati	0.04 (0.76 to 1.41)	0.71 (0.52 to 0.97)	
Brhamin and Kshetri	1.37 (1.00 to 1.88)	0.90 (0.63 to 1.27)	
**Type of place of residence**			0.012
Urban	1	1	
Rural	0.50 (0.38 to 0.67)	0.70 (0.53 to 0.92)	
**Ecological zone**			0.099
Mountain	1	1	
Hill	1.70 (0.94 to 3.06)	1.05 (0.63 to 1.77)	
Terai	1.77 (1.00 to 3.11)	1.47 (0.87 to 2.47)	
**Birth order**			0.000
1	1	1	
2–3	0.50 (0.43 to 0.57)	0.55 (0.46 to 0.66)	
4–5	0.22 (0.17 to 0.28)	0.29 (0.21 to 0.40)	
6+	0.24 (0.15 to 0.37)	0.36 (0.21 to 0.61)	
**Women’s empowerment**			
**Socio-cultural empowerment**			
**Highest educational level**			0.000
No education	1	1	
Primary	1.54 (1.19 to 2.01)	1.33 (1.00 to 1.77)	
Secondary	2.85 (2.23 to 3.64)	1.78 (1.39 to 2.28)	
Higher	5.70 (4.19 to 7.74)	2.08 (1.49 to 2.88)	
**Attitude towards wife beating**			0.167
Negative attitude	1	1	
positive attitude	0.78 (0.64 to 0.94)	0.87 (0.71 to 1.06)	
**Knowledge of property**			0.712
Unknown	1	1	
Known	0.94 (0.69 to 1.27)	0.94 (0.65 to 1.34)	
**Economic Empowerment**			
**Occupation of Women**			0.016
Not working	1	1	
Agriculture	0.63 (0.51 to 0.77)	0.78 (0.62 to 0.97)	
Other occupations	1.57 (1.21 to 2.05)	1.16 (0.87 to 1.54)	
**Property possession**			0.105
Not owned	1	1	
Owned	1.83 (1.41 to 2.37)	1.30 (0.95 to 1.78)	
**Ows bank account**			0.002
No	1	1	
Yes	1.83 (1.51 to 2.21)	1.38 (1.13 to 1.69)	
**Empowerment on media/information technology**			
**Media exposure**			0.002
No access	1	1	
Medium access	2.08 (1.71 to 2.54)	1.38 (1.13 to 1.69)	
High access	3.76 (2.85 to 4.97)	1.56 (1.15 to 2.12)	
**Internet use**			0.000
Never	1	1	
Sometimes	3.78 (3.04 to 4.96)	1.77 (1.42 to 2.21)	
**Familial/interpersonal Empowerment**			
**Decision making power**			0.431
Low	1	1	
High	1.21 (1.04 to 1.41)	1.06 (0.91 to 1.23)	

Media/information technology empowerment was positively associated with skilled delivery services (*p* < 0.005). Women aged 31 and above were more likely to have skilled delivery than younger women (adjusted OR 1.49; 95% CI 1.09 to 2.03). Rural women were less likely to have skilled delivery than their urban counterparts (adjusted OR 0.70; 95% CI, 0.53 to 0.92). The likelihood of having a skilled delivery was higher for women with birth orders of two or three than a first order birth (adjusted OR 0.55; 95% CI, 0.46 to 0.66). Among the three variables in socio-cultural empowerment, educational attainment of the women was significantly positive since highly educated women were more likely to use SBAs (adjusted OR 2.08; 95% CI, 1.49 to 2.88). Similarly, as for economic empowerment, two variables including women who engaged in another occupation (adjusted OR 1.16; 95% CI, 0.87 to 1.54) and having a bank account (adjusted OR 1.38; 95% CI, 1.13 to 1.69) reported high odds. Among the two variables in media and information technology empowerment, both were significant since higher media exposure (adjusted OR 1.56; 95% CI, 1.15 to 2.12) and internet use (adjusted OR 1.77; 95% CI, 1.42 to 2.21) were significantly associated with the utilization of SBAs. However, familial/interpersonal empowerment was not significantly associated with use of SBAs.

We measured the variables of women’s empowerment to confirm the association with SBA utilization without adding any confounding factors. This analysis also provided a similar result as Table 3. Economic empowerment and media and information technology empowerment were more influential than sociocultural empowerment. Specifically, higher education (adjusted OR 2.65; 95% CI, 1.92 to 3.67), an occupation other than agriculture (adjusted OR 1.06; 95% CI, 0.80 to 1.40), owning a bank account (adjusted OR 1.26; 95% CI, 1.04 to 1.53), high access to media (adjusted OR 1.63′ 95% CI, 1.22 to 2.18), and internet use (adjusted OR 2.00; 95% CI, 1.60 to 2.48) were significantly associated with the use of SBAs (Table 4).

**Table 4:** Crude and adjusted Odds Ratios of skilled birth attendance and women’s empowerment using a logistic regression with complex sampling analysis (*n* = 4400)

Variables	Crude OR (95%CI)	Adjusted OR (95%CI)	***P*** value
**Women’s empowerment**			
**Socio-cultural empowerment**			
**Highest educational level**			0.000
No education	1	1	
Primary	1.54 (1.19 to 2.01)	1.41 (1.08 to 1.84)	
Secondary	2.85 (2.23 to 3.64)	2.09 (1.65 to 2.65)	
Higher	5.70 (4.19 to 7.74)	2.65 (1.92 to 3.67)	
**Attitude towards wife beating**			0.135
Negative attitude	1	1	
Positive attitude	0.78 (0.64 to 0.94)	0.86 (0.71 to 1.05)	
**Knowledge of property**			0.843
Unknown	1	1	
Known	0.94 (0.69 to 1.27)	0.97 (0.69 to 1.36)	
**Economic Empowerment**			
**Occupation of Women**			0.000
Not working	1	1	
Agriculture	0.63 (0.51 to 0.77)	0.66 (0.05 to 0.81)	
Other occupations	1.57 (1.21 to 2.05)	1.06 (0.80 to 1.40)	
**Property possession**			0.087
Not owned	1	1	
Owned	1.83 (1.41 to 2.37)	1.30 (0.96 to 1.76)	
**Owns bank account**			0.017
No	1	1	
Yes	1.83 (1.51 to 2.21)	1.26 (1.04 to 1.53)	
**Empowerment on media/information technology**			
**Media exposure**			0.001
No access	1	1	
Medium access	2.08 (1.71 to 2.54)	1.40 (1.14 to 1.73)	
High access	3.76 (2.85 to 4.97)	1.63 (1.22 to 2.18)	
**Internet use**			0.000
Never	1	1	
Sometimes	3.78 (3.04 to 4.96)	2.00 (1.60 to 2.48)	
**Familial/interpersonal Empowerment**			
**Decision making power**			0.627
Low	1	1	
High	1.21 (1.04 to 1.41)	0.96 (0.82 to 1.13)	

We also analyzed the variables of women’s empowerment by separating those living in rural and urban areas. Women having higher education were more likely to use SBAs in both urban (adjusted OR 2.42; 95% CI, 1.59 to 3.67) and rural areas (adjusted OR 2.47; 95% CI, 1.49 to 4.04). Attitude towards wife beating was not significantly associated with using SBAs in urban areas, whereas women having positive attitudes towards wife beating were less likely to have SBAs in rural areas (adjusted OR 0.74; 95% CI, 0.54 to 1.00). Similarly, the occupation of the women was influential in them using SBAs in urban areas (adjusted OR 1.12; 95% CI, 0.78 to 1.60), whereas the occupation of women was not a significant factor in rural areas. Having a bank account in their name was not a significant factor in urban areas, whereas women owning a bank account in their name were more likely to use SBAs in rural areas (adjusted OR 1.59; 95% CI, 1.17 to 2.16). Likewise, media exposure was not significantly associated with using SBAs in urban areas; whereas in rural area, women having high media access were more likely to use SBAs (adjusted OR 1.92; 95% CI, 1.18 to 3.11). Women who sometimes use the internet were more likely to use SBAs in both urban (adjusted OR 2.02; 95% CI, 1.53 to 2.68) and rural areas (adjusted OR 1.88; 95% CI, 1.30 to 2.72) (Table 5).

**Table 5:** Crude and adjusted Odds Ratios of skilled birth attendance in urban and rural areas and women’s empowerment using logistic regression with complex sampling analysis (*n* = 4400)

Urban (***n*** = 2456)				Rural (***n*** = 1944)		
Variables	Crude OR (95%CI)	Adjusted OR (95%CI)	***P*** value	Crude OR (95%CI)	Adjusted OR (95%CI)	***P*** value
**Women’s empowerment**						
**Socio-cultural empowerment**						
**Highest educational level**			0.000			0.000
No education	1	1		1	1	
Primary	1.34 (0.87 to 2.05)	1.24 (0.83 to 1.87)		1.70 (1.19 to 2.43)	1.61 (1.11 to 2.31)	
Secondary	2.24 (1.59 to 3.16)	1.70 (1.23 to 2.36)		3.27 (2.35 to 4.55)	2.51 (1.79 to 3.53)	
Higher	4.89 (3.26 to 7.32)	2.42 (1.59 to 3.67)		4.65 (2.92 to 7.40)	2.47 (1.49 to 4.04)	
**Attitude towards wife beating**			0.728			0.048
Negative attitude	1	1		1	1	
Positive attitude	0.95 (0.76 to 1.20)	1.04 (0.82 to 1.33)		0.66 (0.50 to 0.89)	0.74 (0.54 to 1.00)	
**Knowledge of property**			0.929			0.713
Unknown	1	1		1	1	
Known	0.99 (0.63 to 1.55)	0.98 (0.59 to 1.61)		0.84 (0.55 to 1.29)	0.92 (0.57 to 1.47)	
**Economic Empowerment**						
**Occupation of Women**			0.000			0.142
Not working	1	1		1	1	
Agriculture	0.50 (0.37 to 0.68)	0.60 (0.46 to 0.79)		0.86 (0.63 to 1.16)	0.73 (0.53 to 1.00)	
Other occupations	1.41 (0.99 to 2.01)	1.12 (0.78 to 1.60)		1.44 (0.93 to 2.25)	0.92 (0.62 to 1.39)	
**Property possession**			0.281			0.097
Not owned	1	1		1	1	
Owned	1.75 (1.23 to 2.50)	1.27 (0.82 to 1.95)		1.58 (1.07 to 2.36)	1.43 (0.94 to 2.19)	
**Owns bank account**			0.764			0.004
No	1	1		1	1	
Yes	1.50 (1.17 to 1.92)	1.04 (0.81 to 1.33)		2.05 (1.53 to 2.77)	1.59 (1.17 to 2.16)	
**Empowerment on media/information technology**						
**Media exposure**			0.156			0.013
No access	1	1		1	1	
Medium access	1.93 (1.43 to 2.60)	1.32 (0.96 to 1.82)		1.94 (1.51 to 2.51)	1.33 (1.04 to 1.71)	
High access	3.10 (2.15 to 4.45	1.39 (0.94 to 2.03)		3.86 (2.47 to 6.02)	1.92 (1.18 to 3.11)	
**Internet use**			0.000			0.001
Never	1	1		1	1	
Sometimes	3.35 (2.62 to 4.29)	2.02 (1.53 to 2.68)		3.44 (2.43 to 4.88)	1.88 (1.30 to 2.72)	
**Familial/interpersonal Empowerment**						
**Decision making power**			0.371			0.655
Low	1	1		1	1	
High	1.16 (0.95 to 1.41)	0.91 (0.74 to 1.12)		1.09 (0.87 to 1.37)	0.95 (0.75 to 1.20)	

## Discussion

The empowerment of women was found to be significantly associated with the use of SBAs in our study. In unadjusted regression analysis, education, media exposure and internet use were significantly associated with the use of SBAs. Furthermore, after adjusting the variables of women’s empowerment using sociodemographic characteristics, the variables pertaining to women’s empowerment were still significant. We categorized the variables of women’s empowerment into different dimensions by adopting the concepts of Malhotra et al. [30], Kabeer [26] and the NDHS (2016). Focusing on the categorical dimensions of empowerment, media and information technology empowerment and economic empowerment were found to be more influential than sociocultural and interpersonal/familial empowerment. According to the sensitivity analysis after separating the rural and urban areas, sociocultural empowerment, economic empowerment and media and information technology empowerment were equally influential in urban and rural areas, whereas media and information technology empowerment and sociocultural empowerment were more influential than economic empowerment. However, familial/interpersonal empowerment was not significantly effective in both areas. However, if we emphasize specific variables within the categories, the education of women, occupation, owning a bank account, media exposure, and internet use were significantly associated with the use of SBAs.

### Socio-cultural empowerment

Among the three variables in socio-cultural empowerment, educational attainment was found to be influential in both urban and rural areas as higher educated women reported greater odds of using SBAs. Similar results were reported in other studies in Nepal [11, 34] and elsewhere [7, 12, 35–39]. A possible reason might be that lower educated women lack awareness and tend to follow traditional delivery methods. Education helps change the attitudes of women and widen their knowledge, allowing them to take part in household decision making [40, 41]. The results emphasize the significance of women’s education to achieving MDG 5.

Attitude towards wife beating was not significantly associated with using SBAs in urban areas, but it was significantly associated with using SBAs in rural areas in our study. This result is consistent with other studies [24, 42, 43]; however, they did not analyze rural and urban areas separately. The lack of education and following traditional gender-based misconceptions in rural areas may contribute to supporting wife beating, which hinders access to maternal health care.

### Economic empowerment

Two out of three economic empowerment variables had significant effects in the overall analysis. Women working in non-agricultural sectors were more likely to utilize SBAs than those working in agriculture and not working, which is consistent with other studies [18, 34]. However, women working in non-agricultural sectors were more likely to access maternal health in urban areas, whereas the occupation of women was not influential in rural areas. Another study reported that working women are less likely to obtain skilled deliveries than women not working in Nepal [44]. One possible reason could be linked to the form of work that the women engage in. The rate of women’s engagement in non-agricultural sectors is higher in urban areas than in rural areas. The women who work in areas other than agriculture may earn more money in cash that may empower them to become independent decision makers and use maternal services. Conversely, the majority of women who only work in agriculture in rural areas do not get cash in return to spend it on their health.

The women having bank accounts have a higher propensity of using SBAs. A study in India also documented a significant association of the ownership of a bank account with contraceptive use, ANC visits and greater birth spacing [45]. However, in urban areas, having a bank account in their name did not significantly affect using SBAs, whereas it was influential in rural areas. Owning a bank account might indicate the economic independency and autonomy of women such that they may have freedom in their financial management and health care decisions.

Possession of property in their name was not influential on the use of SBAs in this study. The reason might be that the possession of fixed assets, either solely or jointly, may not empower or induce women to use SBAs unless they have freedom to convert assets into the cash, which is needed to obtain skilled maternal services. The legal possession of property may not mean the freedom to sell and purchase it on their own without the agreement of their partner and other heads of households. Decision making on selling and purchasing fixed assets are usually made by the male head of the household in Nepal.

### Media and information technology empowerment

Another important factor that was positively associated with skilled delivery services was media access. Other studies also documented similar results [22, 23, 46]. However, a study conducted in Ethiopia was inconsistent with our results [7] and showed that media access was not associated with using SBAs. It is consistent with the results in urban areas in our study that access to media was not significantly associated with SBAs. However, women having high access to media were more likely to use SBAs in rural areas. The influence of media might be because media can play crucial roles in bridging knowledge gaps and forming positive attitudes, thereby sensitizing women with the importance of having births using skilled attendants.

Internet access was another predictor of maternal service utilization in both urban and rural areas. A systematic review indicated the importance of internet use during pregnancy since it helps to find information about fatal development, to build the confidence and to encourage decision making [47]. As we have categorized internet use into the media and information technology empowerment of women, internet use might be one of the variables to measure its effectiveness on the use of SBAs. The reason might be that using the internet provides better access to the information that women need when seeking skilled deliveries. In addition, the SNS has become one of the most popular sources of information in Nepal. The positive impact of internet use on the use of SBAs in Nepal might be because of the access to social media via the internet, which allows one to get various information on maternal health services.

### Familial/interpersonal empowerment

Decision making was not influential in both urban and rural areas in this study, which is consistent with the result of a study conducted in Senegal [18]. However, women’s decision making power was significantly associated with using SBAs in other studies in Nepal [16] and elsewhere [6, 8, 11, 12, 19, 22, 24, 48–50]. We did not analyze three aspects (health decisions, movement and larger household purchases) of decision making separately and whether each aspect may have a different effect; rather, we combined them into one variable with two levels. The insignificant relationship between household decision making and the use of SBAs might be questionable because of the uneven distribution of resources and the availability of facilities in all geographical areas in Nepal. Furthermore, as stated by previous researchers, variables pertaining to decision making autonomy might contain multifaceted and similar features as the DHS data [18, 51].

The strength of the study was the use of a national representative sample that is generalizable to the overall scenario of the trend and influencing factors for maternal service utilization. Meanwhile, this study has several limitations. The first is that the definition and operationalization of women’s empowerment introduced in the DHS Nepal lacks contextual relevancy in light of cultural diversification. The second limitation was the cross-sectional design of the study that measured women’s empowerment after the birth of the child. Measuring empowerment before and after the birth may provide a clearer understanding of the influences on SBA use. Third, we selected the variables by limiting the individual information to women only, and adding information on household characteristics and the husband might produce different results.

## Conclusion

The aim of the study was to determine the association between women’s empowerment and the use of SBAs in Nepal. We found a significant association of women’s empowerment with maternal health service utilization. Media and information technology empowerment and economic empowerment were more influential than social-cultural and familial/interpersonal empowerment. However, specifically, educating women, providing and encouraging them to become financially independent and media and information technology access may increase the number of women who seek skilled maternal health services.

## Data Availability

The survey data of this article are freely available at. (URL:https://www.dhsprogram.com/data/dataset/Nepal_Standard-DHS_2016. cfm?) upon making request to ICF, the DHS program.

## Abbreviations

ANC: Antenatal Care
CBS: Central Bureau of Statistics
CSA: Complex Sample Analysis
DHS: Demographic and Health Survey
EAS: Enumeration Areas
GDI: Gender Development Index
GII: Gender Inequality Index
ICF: International Coaching Federation
MDG: Millennium Development Goals
NDHS: Nepal Demographic and Health Survey
NPHS: National Population and Housing Survey
PSUs: Primary Sampling Units
SBA: Skilled Birth Attendants
SDG: Sustainable Development Goals
UN: United Nations

## References

[1] WHO (2019). Trends in maternal mortality: 2000 to 2017: estimates by WHO, UNICEF, UNFPA, World Bank Group and the United Nations Population Division Geneva, Licence: CC BY-NC-SA 3.0 IGO.

[2] WHO (2011). Millennium development goals: progress towards the health- related millennium development goals; improved maternal health (mdg 5), fact sheet.

[3] Bullough C, Meda N, Makowiecka K, Ronsmans C, Achadi EL, Hussein J (2005). Current strategies for the reduction of maternal mortality. BJOG.

[4] Ministry of Health NNE, ICF. a (2017). Nepal Demographic and Health Survey 2016.

[5] CBS (2012). National population and housing census.

[6] Zakar R, Zakar MZ, Aqil N, Chaudhry A, Nasrullah M (2017). Determinants of maternal health care services utilization in Pakistan: evidence from Pakistan demographic and health survey, 2012-13. J Obstet Gynaecol.

[7] Amoakoh-Coleman M, Ansah EK, Agyepong IA, Grobbee DE, Kayode GA, Klipstein-Grobusch K (2015). Predictors of skilled attendance at delivery among antenatal clinic attendants in Ghana: a cross-sectional study of population data. BMJ Open.

[8] Tarekegn SM, Lieberman LS, Giedraitis V (2014). Determinants of maternal health service utilization in Ethiopia: analysis of the 2011 Ethiopian demographic and health survey. BMC Pregnancy Childbirth.

[9] Prata N, Tavrow P, Upadhyay U (2017). Women’s empowerment related to pregnancy and childbirth: introduction to special issue. BMC Pregnancy Childbirth.

[10] UN. Transforming our world: The 2030 Agenda for Sustainable Development. New York; 2015. Available from: https://sustainabledevelopment.un.org. 27 Sept 2019.

[11] Furuta M, Salway S (2006). Women’s position within the household as a determinant of maternal health care use in Nepal. Int Fam Plan Perspect.

[12] Shimamoto K, Gipson JD (2019). Investigating pathways linking women's status and empowerment to skilled attendance at birth in Tanzania: a structural equation modeling approach. PLoS One.

[13] Phan L (2016). Measuring Women’s empowerment at household level using DHS data of four southeast Asian countries. Soc Indic Res.

[14] Miedema SS, Haardörfer R, Girard AW, Yount KM (2018). Women’s empowerment in East Africa: development of a cross-country comparable measure. World Dev.

[15] Ewerling F, Lynch JW, Victora CG, van Eerdewijk A, Tyszler M, Barros AJD (2017). The SWPER index for women's empowerment in Africa: development and validation of an index based on survey data. Lancet Glob Health.

[16] Kc S, Neupane S (2016). Women's autonomy and skilled attendance during pregnancy and delivery in Nepal. Matern Child Health J.

[17] Sebayang SK, Ferry Efendi A, Astutik E (2017). Women’s Empowerment and the Use of Antenatal Care Services in Southeast Asian Countries.

[18] Shimamoto K, Gipson JD (2017). Examining the mechanisms by which women’s status and empowerment affect skilled birth attendant use in Senegal: a structural equation modeling approach. BMC Pregnancy Childbirth..

[19] Chol C, Negin J, Agho KE, Cumming RG (2019). Women’s autonomy and utilisation of maternal healthcare services in 31 sub-Saharan African countries: results from the demographic and health surveys, 2010-2016. BMJ Open.

[20] Izudi J, Akwang DG, McCoy SI, Bajunirwe F, Kadengye DT (2019). Effect of health education on birth preparedness and complication readiness on the use of maternal health services: a propensity score-matched analysis. Midwifery..

[21] Asaolu IO, Alaofè H, JKL G, Adu AK, Monroy AJ, Ehiri JE (2018). Measuring women's empowerment in Sub-Saharan Africa: exploratory and confirmatory factor analyses of the demographic and health surveys. Front Psychol.

[22] Singh PK, Singh L (2014). Examining inter-generational differentials in maternal health care service utilization: insights from the Indian demographic and health survey. J Biosoc Sci.

[23] Mezmur M, Navaneetham K, Letamo G, Bariagaber H (2017). Individual, household and contextual factors associated with skilled delivery care in Ethiopia: evidence from Ethiopian demographic and health surveys. PLoS One.

[24] Sado L, Spaho A, Hotchkiss DR (2014). The influence of women’s empowerment on maternal health care utilization: evidence from Albania. Soc Sci Med.

[25] UN (2019). Human Development Report 2019: Beyond income, beyond averages, beyond today: Inequalities in human development in the 21st century.

[26] Kabeer N (2005). Gender equality and women’s empowerment: a critical analysis of the third millennium development goal 1. Gend Dev.

[27] Lee-Rife SM (2010). Women’s empowerment and reproductive experiences over the lifecourse. Soc Sci Med.

[28] Mosedale S (2005). Assessing women’s empowerment: towards a conceptual framework. J Int Dev.

[29] Tengland PA (2008). Empowerment: a conceptual discussion. Health Care Anal.

[30] Malhotra A, Schuler SR, Boender C (2002). Measuring women’s empowerment as a variable in international development. background paper prepared for the World Bank Workshop on Poverty and Gender: New Perspectives.

[31] Kishor S, Subaiya L (2008). Understanding Women’s Empowerment: A Comparative Analysis of Demographic and Health Surveys (DHS) Data. DHS Comparative Reports No. 20.

[32] WHO (2006). Reproductive Health Indicators : Reproductive Health and Research Guidelines for their generation, interpretation and analysis for global monitoring WHO Press.

[33] ICF International (2012). Survey organization manual for demographic and health surveys. MEASURE DHS.

[34] Neupane S, Doku D (2013). Utilization of postnatal care among Nepalese women. Matern Child Health J.

[35] Prusty RK, Gouda J, Pradhan MR. Inequality in the Utilization of Maternal Healthcare Services in Odisha, India. Int J Popul Res. 2015;2015:531485.

[36] Aliy J, Mariam DH (2012). Determinants of equity in utilization of maternal health services in Butajira, southern Ethiopia. Ethiop J Health Dev.

[37] Fekadu M, Regassa N (2014). Skilled delivery care service utilization in Ethiopia: analysis of rural-urban differentials based on national demographic and health survey (DHS) data. Afr Health Sci.

[38] Sagna ML, Sunil T (2012). Effects of individual and neighborhood factors on maternal care in Cambodia. Health Place.

[39] Singh PK, Kumar C, Rai RK, Singh L (2013). Factors associated with maternal healthcare services utilization in nine high focus states in India: a multilevel analysis based on 14 385 communities in 292 districts. Health Policy Plan.

[40] Caldwell JC. Education as a Factor in Mortality Decline An Examination of Nigerian Data. Popul Stud. 1979;33(3):395–413.

[41] Jejeebhoy SJ. Women's education, autonomy, and reproductive behaviour: Experience from developing countries. OUP Catalogue. New York: Oxford University Press; 1995.

[42] Haque SE, Rahman M, Mostofa MG, Zahan MS (2012). Reproductive health care utilization among young mothers in Bangladesh: does autonomy matter?. Women’s Health Issues.

[43] Corroon M, Speizer IS, Fotso JC, Akiode A, Saad A, Calhoun L (2014). The role of gender empowerment on reproductive health outcomes in urban Nigeria. Matern Child Health J.

[44] Matsumura M, Gubhaju B (2001). Women’s status, household structure and the utilization of maternal health services in Nepal. Asia-Pac Popul J.

[45] Singh A, Kumar K, McDougal L, Silverman JG, Atmavilas Y, Gupta R (2019). Does owning a bank account improve reproductive and maternal health services utilization and behavior in India? Evidence from the National Family Health Survey 2015–16. SSM Popul Health.

[46] Babalola S, Fatusi A (2009). Determinants of use of maternal health services in Nigeria - looking beyond individual and household factors. BMC Pregnancy Childbirth..

[47] Javanmardi M, Noroozi M, Mostafavi F, Ashrafi-Rizi H (2018). Internet usage among pregnant women for seeking health information: a review article. Iran J Nurs Midwifery Res.

[48] Ahmed S, Creanga AA, Gillespie DG, Tsui AO (2010). Economic status, education and empowerment: implications for maternal health service utilization in developing countries. PLoS One.

[49] Mistry R, Galal O, Lu M (2009). Women’s autonomy and pregnancy care in rural India: a contextual analysis. Soc Sci Med.

[50] Rizvi N, S Khan K, Shaikh BT (2014). Gender: shaping personality, lives and health of women in Pakistan. BMC Women's Health.

[51] Koblinsky M, Matthews Z, Hussein J, Mavalankar D, Mridha M, Anwar I (2006). Maternal survival 3 - going to scale with professional skilled care. Lancet..

